# Hepatitis B vaccine delivered by microneedle patch: Immunogenicity in mice and rhesus macaques

**DOI:** 10.1016/j.vaccine.2023.05.005

**Published:** 2023-05-11

**Authors:** Youkyung Choi, Grace Sanghee Lee, Song Li, Jeong Woo Lee, Tonya Mixson-Hayden, Jungreem Woo, Dengning Xia, Mark R. Prausnitz, Saleem Kamili, Michael A. Purdy, Rania A. Tohme

**Affiliations:** a Division of Viral Hepatitis, National Center for HIV, Viral Hepatitis, STD and TB Prevention, US Centers for Disease Control and Prevention (CDC), Atlanta, GA, USA; b School of Chemical and Biomolecular Engineering, Georgia Institute of Technology, Atlanta, GA, USA; c Global Immunization Division, Centers for Global Health, CDC, Atlanta, GA, USA

**Keywords:** Hepatitis B vaccine, Birth dose vaccination, Microneedle patch delivery, Cellular and adaptive immune responses, Host gene expression profile to vaccination

## Abstract

Vaccination against hepatitis B using a dissolving microneedle patch (dMNP) could increase access to the birth dose by reducing expertise needed for vaccine administration, refrigerated storage, and safe disposal of biohazardous sharps waste. In this study, we developed a dMNP to administer hepatitis B surface antigen (HBsAg) adjuvant-free monovalent vaccine (AFV) at doses of 5 μg, 10 μg, and 20 μg, and compared its immunogenicity to vaccination with 10 μg of standard monovalent HBsAg delivered by intramuscular (IM) injection either in an AFV format or as aluminum-adjuvanted vaccine (AAV). Vaccination was performed on a three dose schedule of 0, 3, and 9 weeks in mice and 0, 4, and 24 weeks in rhesus macaques. Vaccination by dMNP induced protective levels of anti-HBs antibody responses (≥10 mIU/ml) in mice and rhesus macaques at all three HBsAg doses studied. HBsAg delivered by dMNP induced higher anti-HBsAg antibody (anti-HBs) responses than the 10 μg IM AFV, but lower responses than 10 μg IM AAV, in mice and rhesus macaques. HBsAg-specific CD4+ and CD8+ T cell responses were detected in all vaccine groups. Furthermore, we analyzed differential gene expression profiles related to each vaccine delivery group and found that tissue stress, T cell receptor signaling, and NFκB signaling pathways were activated in all groups. These results suggest that HBsAg delivered by dMNP, IM AFV, and IM AAV have similar signaling pathways to induce innate and adaptive immune responses. We further demonstrated that dMNP was stable at room temperature (20 °C–25 °C) for 6 months, maintaining 67 ± 6 % HBsAg potency. This study provides evidence that delivery of 10 μg (birth dose) AFV by dMNP induced protective levels of antibody responses in mice and rhesus macaques. The dMNPs developed in this study could be used to improve hepatitis B birth dose vaccination coverage levels in resource limited regions to achieve and maintain hepatitis B elimination.

## 1. Introduction

Globally, 296 million persons are living with chronic hepatitis B virus (HBV) infection and nearly 820,000 deaths occurred in 2019 as a result of HBV-related liver cirrhosis and hepatocellular carcinoma [1]. Perinatal and early childhood HBV infection is a major risk factor for developing chronic HBV infection, as 50–80 % of infants infected before the age of 5 years develop chronic HBV infection [2,3]. The World Health Organization (WHO) recommends universal vaccination with a hepatitis B vaccine birth dose (HepB-BD) within the first 24 h of life (timely HepB-BD), followed by timely completion of 2–3 additional doses of hepatitis B vaccine to prevent mother-to-child and horizontal transmission of HBV [4,5].

In 2016, the World Health Assembly endorsed the Global Health Sector Strategy on Viral Hepatitis, which calls for elimination of hepatitis B, including elimination of mother to child transmission (MTCT) of hepatitis B by 2030 [6]. Elimination of HBV infection requires achieving a prevalence of hepatitis B surface antigen (HBsAg) at ≤0.1 % in children 5 years of age and achievement of ≥90 % coverage with timely HepB-BD and 3 doses of hepatitis B vaccine (HepB3) [7]. A timely HepB-BD, administered within 24 h of birth, is essential to prevent MTCT and can be a critical tool to achieve the HBV elimination goal. Despite years of availability, global coverage with HepB-BD has been approximately 42% [8]. While the Africa Region has a high prevalence of chronic HBV infection in children globally, timely HepB-BD coverage was only 17 % in 2021 [8]. In resource-limited settings where over half of the children are born outside health facilities, some barriers to introduction of and improving coverage with HepB-BD include high rates of home births and deliveries by unskilled birth attendants [9,10]. In addition, lack of a cold chain hinders HepB-BD vaccination in some settings [5,11].

The standard method of administration of HepB-BD is intramuscular (IM) injection, which requires experienced health care staff. Additionally, injections generate biohazardous sharps waste that requires safe disposal; mishandling of needles and syringes might result in sharps injuries and transmission of bloodborne pathogens [12]. Therefore, safe and effective needle-free vaccination is needed as an alternative to conventional needle-and-syringe vaccination. To address these needs, we and others have developed dissolving microneedle patches (dMNP) that can be administered by manual application to the skin with little or no training, can be formulated for thermostability for storage without refrigeration, and generate no biohazardous sharps waste because the microneedles dissolve in the skin [13–15]. Such a dMNP could fulfill public health needs in improving HepB-BD coverage. A dMNP patch for influenza vaccination was well tolerated and generated good antibody responses in a phase 1 clinical trial [16], and measles and rubella dMNP vaccines have also generated protective immune responses in rhesus macaques [17,18], and are the subject of an on-going phase 1/2 clinical trial [19]. Recently, dMNPs containing a combination of polio and rotavirus vaccine elicited good immunogenic responses [20].

In our previous study, HepB vaccination delivered by dMNP induced seroprotective levels of antibodies (≥10 mIU/ml) to hepatitis B surface antigen (anti-HBs) in mice and rhesus macaques [21,22]. In the current study, we build on those findings to further develop the dMNPs to improve dosing control and to examine cellular and humoral immune responses in vaccinated mice and rhesus macaques. In addition, the host gene expression profiles of vaccine reactogenicity in each vaccination group (dMNP and IM injection) were identified.

## 2. Methods

### 2.1. Hepatitis B vaccine

Bulk solution of aluminum adjuvant-free monovalent hepatitis B vaccine (AFV HBsAg) was kindly provided by the Serum Institute of India (Pune, India). Hepatitis B vaccine consists of purified surface antigen of HBV expressed in the yeast cells (*Hansenula polymorpha*) [23]. The starting antigen concentration was 1.63 mg/mL that was further concentrated to 14 mg/ml for dMNP fabrication by centrifugal filtration (Amicon Ultra-0.5 ml, MWCO = 3 K Da, Millipore Sigma, Burlington, MA). In addition, the bulk HBsAg was used for preparation of adjuvant-free monovalent hepatitis B vaccine (AFV, 10 μg/ml) for intramuscular (IM) injection. Aluminum-adjuvanted monovalent hepatitis B vaccine (AAV) (ENGERIX-B, 10 μg/0.5 ml) for IM injection was purchased from GlaxoSmithKline (Research Triangle Park, NC).

### 2.2. Selection of optimal formulation of dMNP fabrication and assessment of HBsAg dose delivery

The AFV was mixed with 5 % (w/v) of different stabilizers (sorbitol, xylitol, glucose, maltose, sucrose and trehalose) (Sigma, St. Louis, MO) in either phosphate-buffered saline (PBS) or Hank’s balanced salt solution (HBSS) buffer. A 50 μl formulation mixture was dried on a polydimethylsiloxane (PDMS) sheet at room temperature (20 – 25 °C) for 1 day. The dried mixture was reconstituted in 100 μl PBS and diluted 2000-fold for HBsAg ELISA analysis (RecombiLISA, CTK Bioteck, Poway, CA) to determine HBsAg potency.

### 2.3. Dissolving microneedle patch (dMNP) fabrication

dMNPs were made in a two-step molding process on PDMS (Sylgard 184, Dow Corning, Midland, MI) molds based on an established method [21,22] with minor modification, as described below. The molds contained 100 conical cavities (i.e., to form the dMNPs) arranged in a 10-by-10 array in an area of 0.5 cm^2^. Each dMNP cavity comprised a microneedle of 600 μm in length with a 200 μm base diameter and a ~10 μm radius of curvature at the tip. At the opening of each microneedle cavity was a tapered “funnel” region measuring 400 μm in length and ~600 μm diameter in opening that facilitated mold filling during fabrication and produced a pedestal on which each microneedle was mounted in the resulting dMNP.

Briefly, AFV was added into a first casting solution containing 7 % (w/v) maltodextrin (dextrose equivalent 13.0–17.0, Sigma-Aldrich, St. Louis, MO) and 3 % (w/v) sucrose (Sigma-Aldrich) in PBS (Omnipur, Baltimore, MD) that was cast on molds under vacuum for 30 min to form the dMNPs. Excess solution on the mold surface was removed, and a second solution containing 30 % (w/v) fish gelatin (cold water fish skin, Sigma-Aldrich) and 20 % (w/v) sucrose in PBS was cast on the mold to form the backing layer of the dMNPs. The filled mold was kept under vacuum for another 3 h and then further dried in a desiccator at room temperature for 3 days. After demolding, all dMNPs were sealed in individual pouches with desiccant and stored at 4 °C until use. Patches were allowed to return to room temperature in their pouches prior to application to animals. For initial *in vitro* testing of HBsAg delivery, two different doses (10 μg and 20 μg) of dMNPs were prepared and tested for delivery dosage by *in vitro* insertion as described previously, respectively [21]. In order to deliver 5 μg, 10 μg, and 20 μg of HBsAg doses, 10 μg, 20 μg, and 40 μg of HBsAg were encapsulated in dMNPs, based on an expected delivery efficiency of ~50 %.

### 2.4. Delivery of dMNP vaccine in mice

The animal use protocol (protocol 3181CHOMONC) and procedures were reviewed and approved by the Institutional Animal Care and Use Committees of the US Centers for Disease Control and Prevention (CDC) and Georgia Institute of Technology. All animal experiments were complied with the National Research Council’s Guide for the Care and Use of Laboratory Animals. The backs of mice receiving dMNP vaccination were shaved with electric shears followed by application of a depilatory cream (Nair, Church & Dwight, Ewing, NJ) before vaccination. The mice were anesthetized by intraperitoneal injection of 100 μl PBS containing ketamine (110 mg/kg) and xylazine (11 mg/kg) before applying the dMNP. Thirty-two 11-week-old female BALB/c mice (Charles River, Wilmington, MA) were immunized by dMNP vaccine or intramuscular (IM) injection with 10 μg AFV in four groups: approximately 5 μg (n = 8), 10 μg (n = 8) or 20 μg (n = 8) by dMNP, or 10 μg (n = 8) by IM injection. dMNPs were manually applied to the dorsal skin of the mice and left in place for 20 min to allow microneedle dissolution in the skin. Vaccination was performed at 0, 3, and 9 weeks.

### 2.5. Delivery of dMNP vaccine in rhesus macaques

Each rhesus macaque was placed in ventral recumbency and sedated with ketamine (10 mg/kg) or telazol (4 mg/kg) administered by IM injection for vaccination. The back of the rhesus macaques was shaved with electric shears and depilatory lotion (Nair, Church & Dwight) was used for removing hair for dMNP delivery. Application of dMNPs was performed as described before [22]. Briefly, dMNPs were manually applied to the skin for 10 sec with pressure to the center of the patch to facilitate microneedle insertion and held for an additional 50 sec to confirm the tight contact of the patches to the skin. Then, the dMNP remained in place, undisturbed for 15 min before removal. Intramuscular vaccine injections to the vastus lateralis muscle on the leg were performed using a 26-gauge ½-inch hypodermic needle and syringe. A total of 20 rhesus macaques (10 female, 10 male, age 19 months-2 years old, 2.6 kg to 5.1 kg, Alpha Genesis, Yemassee, SC) were vaccinated in 5 groups as follows: AFV delivered at a dose of approximately 5 μg (n = 4), 10 μg (n = 4) or 20 μg (n = 4) by dMNP, and for IM delivery, 10 μg of HepB was delivered either as AFV (n = 4), or AAV (n = 4). The animals in each of the 5 groups were given 3 doses of hepatitis B vaccine at 0, 4, and 24 weeks (Table 1).

### 2.6. Blood samples

For blood collection, mice were anesthetized in an induction chamber charged with 5 % isoflurane in O_2_ by isoflurane vaporizer (SurgiVet Model 100, Smiths Medical, Minneapolis, MN), and then fitted with a standard rodent mask and kept under general anesthesia by setting the vaporizer at 1–2 % isoflurane flow during the procedures. For rhesus macaques, Ketamine (4 mg/kg) was used. The anesthetized rhesus macaques were reversed with atipamezole (0.02 mg/kg). Blood specimens were collected before the first dose of hepatitis B vaccine. After the initial vaccination, blood samples were collected biweekly until week 16 in mice and weekly until week 44 in rhesus macaques. Whole blood samples were collected using CPT tubes (BD Vacutainer, Becton Dickinson, Franklin Lakes, NJ), and peripheral blood mononuclear cells (PBMCs) were separated according to the manufacturer’s protocol (Becton Dickinson). Recovery cell culture freezing medium (Gibco, Pittsburgh, PA) was added to the PBMCs before storage in liquid nitrogen until use.

### 2.7. Analysis of anti-HBs responses

Anti-HBs antibody responses to dMNP vaccination in mice were compared with IM delivery of 10 μg (pediatric dose) of AFV. Levels of anti-HBs responses were measured by mouse anti-HBs antibody ELISA kit according to the manufacturer’s protocol (mouse anti-HBs AB ELISA, LifeSpan BioSciences, Seattle, WA). For rhesus macaques, dMNP delivery of 5, 10, and 20 μg HepB was compared with IM injection of 10 μg of AFV and AAV. Levels of anti-HBs responses were measured by human anti-HBs antibody ELISA kit according to the manufacturer’s protocol (RecombiLISA, CTK Bioteck, Poway, CA). Anti-HBs levels ≥10 mIU/mL are considered seroprotective against HBV infection in immunocompetent adults and children [24].

### 2.8. Analysis of vaccine-specific T cell responses

Frequency of HBsAg-specific CD4+ and CD8+ T cells was analyzed with flow cytometric analysis. Cryopreserved PBMCs were thawed in a 37 °C water bath and washed with PBS. Cells were counted and seeded 1 × 10^6^ cells per well in 96-well U-bottom plates (Corning, Durham, NC). To block non-specific Fc-mediated interaction, 2.5 μg of Fc block (Fc Block, BD Biosciences, Franklin Lakes, NJ) was added to the cells and incubated for 10 min at room temperature. Monoclonal antibodies used for staining of PBMCs were PerCP-Cy5.5-anti CD4 (#552838) and FITC-anti-CD8 (#555366) from BD Biosciences. Antibody staining was performed at 4 °C for 30 min. HBsAg-specific CD4+ and CD8+ cells were sorted using allophycocyanin (APC) labeled HBsAg. After extensive washing, the stained cells were analyzed by BD Accuri C6 Plus (BD Biosciences) and FlowJo version 10.7.1 (Tree Star, Ashland, OR).

### 2.9. RNA extraction and NanoString nCounter gene expression analysis

Immune response profiles were analyzed to determine reactogenicity induced by dMNP or IM vaccination. Total RNA was extracted from PBMC samples obtained from rhesus macaques using RiboPure RNA purification kit (Thermo Fisher Scientific, Pittsburgh, PA) according to the manufacturer’s instructions. Quality and quantity of extracted RNA was analyzed by a 2100 bioanalyzer (Agilent, Santa Clara, CA). Total RNA of 100 ng from each sample was hybridized using the Host Response Profiling Panel of 785 genes (cat# 115000495) on the nCounter system (NanoString Technologies, Seattle, WA) for 16 h at 65 °C, followed by incubation at 4 °C for up to 8 h. Differential gene expression and identification of enriched pathways were analyzed with ROSALIND software version 3.35.12.0 (Rosalind, San Diego, CA). Normalization, fold changes, and *P*-values were calculated using criteria provided by Nanostring. The fold change of each gene was calculated as the ratio of the average gene expression in each vaccination group to that of the baseline sample (before vaccination). Significantly regulated genes were selected with fold change ≥1.5 or ≤−1.5 and a *P*-value ≤ 0.05. *P*-value adjustment was performed using the Benjamini-Hochberg method of estimating false discovery rates (FDR). Multidimensional Scaling (MDS) graphs were generated for individual samples using ROSALIND software. Gene-set enrichment analysis (GSEA) was performed using databases including Interpro [25], NCBI [26], MSigDB [27], REACTOME [28], and WikiPathways [29] and calculated relative to a set of genes in the baseline relevant for vaccination samples. Clustering of genes for the final heatmap of differentially expressed genes was visualized by Prism 9.3.1 (GraphPad, San Diego, CA).

### 2.10. Statistical analysis

Statistical analyses were performed in Prism 9.3.1 (GraphPad). Two-way ANOVA test was performed to analyze *P*-values of HBsAg recovery, anti-HB responses, and CD4+/CD8+ T cell responses in different vaccination groups. *P*-values ≤ 0.05 were considered significant.

## 3. Results

### 3.1. dMNP formulation, delivery efficiency, and stability

**Fig. 1.**
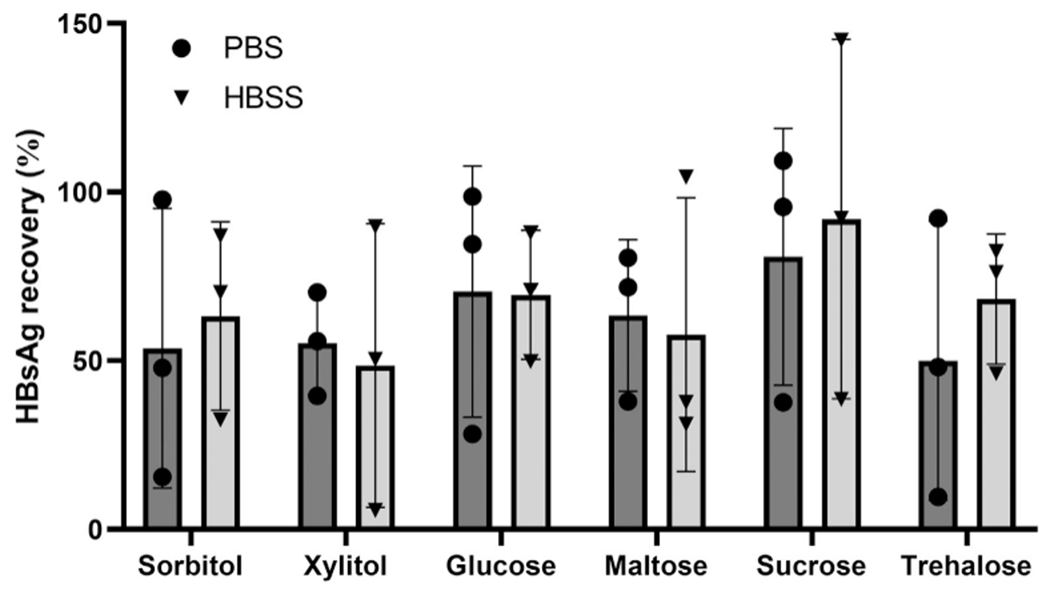
Formulation for dMNP for HBsAg delivery. Adjuvant-free hepatitis B vaccine (40 μg/ml) was mixed with one of six different stabilizers (sorbitol, xylitol, glucose, maltose, sucrose, and trehalose) in either phosphate-buffered saline (PBS, •) or Hank’s balanced salt solution (HBSS, ▼). Each formulation was cast onto a polydimethylsiloxane (PDMS) surface, dried, and reconstituted in PBS before analyzing HBsAg levels by ELISA. Data shown mean values ± standard deviation.

Because dMNP fabrication involves casting and drying HBsAg onto a PDMS mold, we first screened twelve casting solution formulations varying in stabilizer and buffer types to determine which ones provided optimal stability. AFV was formulated with six different stabilizers (sorbitol, xylitol, glucose, maltose, sucrose, and trehalose) in either PBS or HBSS. After casting, drying, and reconstituting in PBS we found that there was no significant difference in the percent recovery of HBsAg as a function of formulation, with all yielding approximately 49 % to 92 % recovery (Fig. 1). Although not statistically significantly different among the different stabilizers, the highest levels of HBsAg were recovered from dMNPs formulated with sucrose, so we elected to use the sucrose/PBS formulation for dMNP fabrications. HBsAg delivery efficiency of dMNPs was tested by measuring antigen content in dMNPs before and after insertion into porcine skin *ex vivo*. dMNPs containing 21.3 ± 3 μg or 39.8 ± 5.3 μg were found to deliver 10.1 ± 1.5 μg or 21.2 ± 2.2 μg, respectively (Supplemental Fig. 1a), which corresponded a delivery efficiency of 48.6 ± 10 % or 54.5 ± 11.2 %, respectively (Supplemental Fig. 1b). dMNPs stored at room temperature for 6 months. HBsAg levels in dMNPs were measured by ELISA kit and found that retained 67 ± 6 % of the initial HBsAg dose (Supplemental Fig. 1c).

### 3.2. Immunogenicity of dMNP vaccination in mice

Next, we determined the immunogenicity of HBsAg administered by dMNP compared to IM injection in mice. By measuring HBsAg in dMNPs before and after administration, we found that the dMNPs designed to deliver 5 μg, 10 μg, or 20 μg of HBsAg actually delivered on average of 6.4 ± 2.8 μg, 8.3 ± 2.2 μg, or 18.3 ± 7.4 μg of HBsAg after the first dose and 7.1 ± 2.0 μg, 5.2 ± 2.6 μg, or 21.9 ± 4.9 μg of HBsAg after the second dose, respectively (Supplemental Fig. 2b). dMNPs used for the 3rd dose were not available for the dose characterization. Vaccination by dMNP was compared to IM vaccination of 10 μg AFV.

**Fig. 2.**
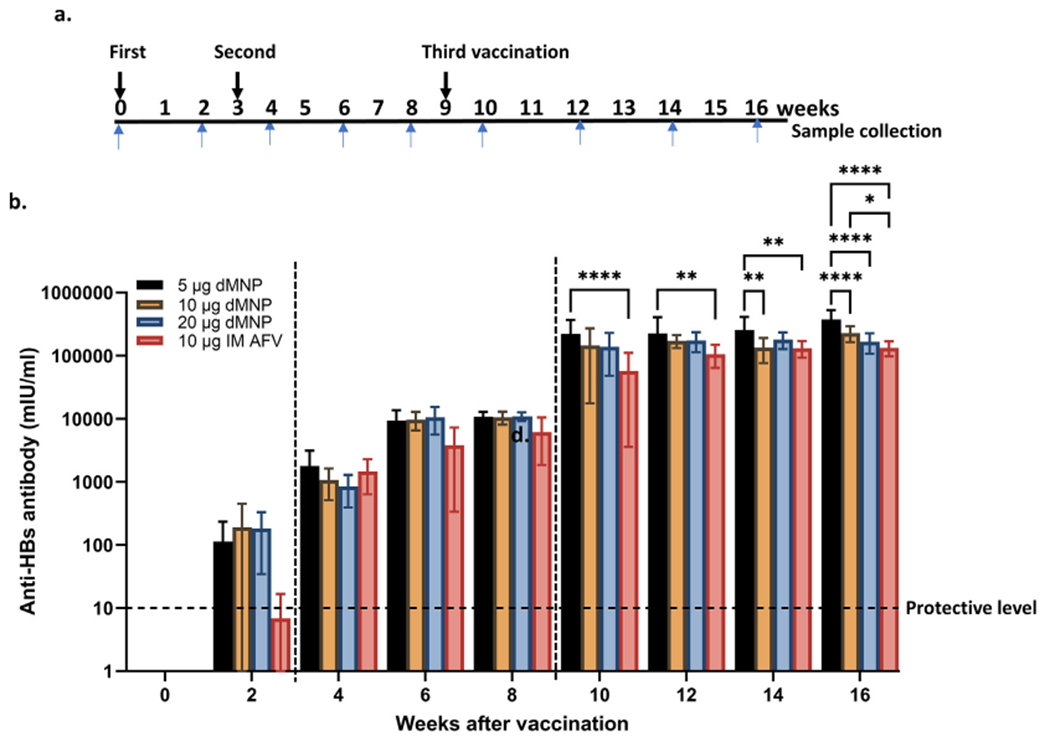
Humoral immunogenicity of HBsAg administered by dMNP to mice. (**a**) Vaccination schedule. Three doses were administered at 0, 3, and 9 weeks. Four vaccination groups [5 μg (n = 8), 10 μg (n = 8), 20 μg (n = 8) dose dMNPs, and 10 μg AFV (n = 8) by intramuscular injection] were used. Downward pointing arrows indicate first (red), second (green), and third (dark blue) vaccine dose, and bi-weekly sample collection (upward pointing arrows, light blue). (**b**) Anti-HBs antibody responses in each vaccination group. Horizonal dotted line indicates protective levels of anti-HBs antibody (≥10 mIU/ml) and vertical dotted lines indicate vaccination at 3 weeks and 9 weeks. Two-way ANOVA test was performed to analyze *P* values in different vaccination groups. * *P* ≤ 0.05, ** *P* ≤ 0.01, *** *P* ≤ 0.001, **** *P* ≤ 0.0001. Data shown mean values ± standard deviation. (For interpretation of the references to colour in this figure legend, the reader is referred to the web version of this article.)

Mice receiving 5 μg, 10 μg, or 20 μg HBsAg by dMNPs had anti-HBs antibody levels ≥10 mIU/ml (range 18 to 814 mIU/ml) at 2 weeks after the first dose (Fig. 2). Mice receiving 10 μg AFV group by IM injection also had anti-HBs levels ≥10 mIU/ml (range 1.4 to 30.5 mIU/ml). After the 2nd and 3rd vaccine doses, all vaccinated animals showed significant increases in anti-HBs levels in which all mice had levels ≥10 mIU/ml. At 4 weeks (i.e., 1 week after the 2nd dose), average anti-HBs levels were 1,789 mIU/ml, 1074 mIU/ml, 841 mIU/ml in 5 μg, 10 μg, and 20 μg dMNP vaccination groups, respectively. Anti-HBs levels at 10 weeks (i.e., 1 week after the 3rd dose) were 22,134 mIU/ml, 1,44,687 mIU/ml, and 1,38,636 mIU/ml in 5 μg, 10 μg, and 20 μg dMNP vaccination groups, respectively. Mice receiving 10 μg AFV IM had anti-HBs levels of 1,463 mIU/ml and 57,039 mIU/ml at 4 and 10 weeks, respectively.

Anti-HBs levels in dMNP vaccination groups were not significantly different from each other, except for anti-HBs responses of the 5 μg dMNP group, which were higher compared with the 10 μg dMNP group at 14 (*P* = 0.0014) and 16 weeks (*P* < 0.0001), and the 20 μg dMNP group at 16 weeks (*P* < 0.0001). Anti-HBs responses of the 10 μg IM AFV group were 1.9 to 3.9-fold lower compared with the 5 μg dMNP group at 10 weeks (*P* < 0.0001), 12 weeks (*P* = 0.0015), 14 weeks (*P* = 0.0011), and 16 weeks (*P* < 0.0001), and the 10 μg dMNP group at 16 weeks (*P* = 0.0203), respectively (Fig. 2).

### 3.3. Immunogenicity of dMNP vaccination in rhesus macaques

We next conducted a study of dMNP vaccination in rhesus macaques. In this case, the dMNPs designed to deliver 5 μg, 10 μg, or 20 μg HBsAg and actually administered an average of 7.6 ± 0.7 μg to 8.9 ± 0.3 μg, 14.9 ± 2.6 μg to 16.5 ± 0.7 μg, and 19 ± 0.9 μg 30 ± 4.6 μg, respectively (Supplemental Fig. 3). Vaccination by dMNP was compared to IM vaccination of 10 μg AFV and IM vaccination of 10 μg AAV.

**Fig. 3.**
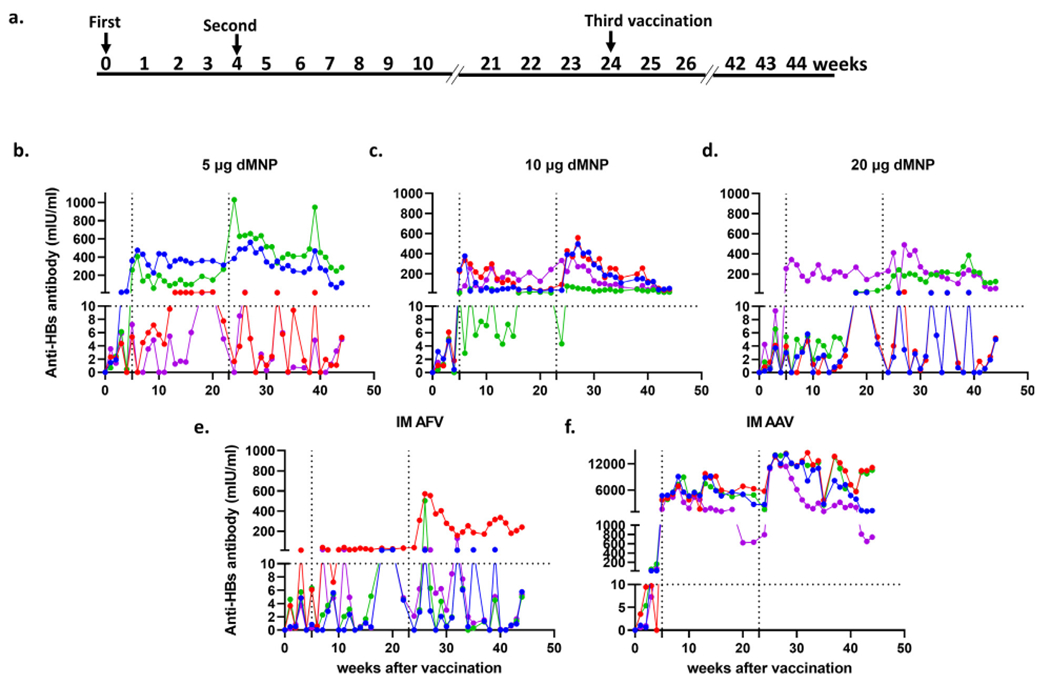
Humoral immunogenicity of HBsAg administered by dMNP to rhesus macaques. (**a**) Vaccination schedule. Three doses were administered at 0, 4, and 24 weeks. Anti-HBs antibody levels after dMNP vaccination with adjuvant-free HBsAg at a dose of approximately 5 μg (**b**), 10 μg (**c**), and 20 μg (**d**); intramuscular (IM)-injection of adjuvant-free HBsAg (IM AFV) (**e**); IM injection of standard aluminum-adjuvanted HBsAg (IM AAV) (**f**). Anti-HBs levels were analyzed in weekly serum samples following vaccination. Data points indicate titers for each animal in each experimental group (n = 4 per group). Seropositive titers were defined as any detectable anti-HBs level, and the protective threshold of ≥ 10 mIU/mL is shown by the horizontal dotted line. Vertical dotted lines indicate vaccinations at 4 weeks and 24 weeks.

Three weeks after the first dose, protective levels of anti-HBs responses (≥10 mIU/ml) were detected in two of the four animals receiving IM 10 μg AAV (Fig. 3b). Anti-HBs levels increased significantly in all vaccinated animals after the second dose. Three macaques receiving 5 μg dMNP, four receiving 10 μg dMNP, two receiving 20 μg dMNP, two receiving IM AFV and four receiving IM AAV had anti-HBs levels ≥ 10 mIU/ml at 16 weeks after the first dose. After the third dose, anti-HBs responses were increased in two macaques receiving 5 μg dMNP and anti-HBs levels fluctuated in the other two macaques. At the end of observation, two macaques receiving 5 μg dMNP had anti-HBs levels ≥10 mIU/ml (range 5.3 mIU/ml to 287 mIU/ml). The third dose induced increase in anti-HBs responses in three of four macaques receiving 10 μg dMNP and all four macaques had anti-HBs levels ≥10 mIU/ml (range 16 to 54 mIU/ml) at the end of observation. Anti-HBs responses increased in two macaques receiving 20 μg dMNP after the 3rd dose and two macaques had anti-HBs levels ≤10 mIU/ml (range 5 to 124 mIU/ml) at the end of observation. Anti-HBs responses were increased in one macaque receiving IM AFV after the 3rd dose and one macaque had anti-HBs AB ≥10 mIU/ml (range 5 mIU/ml to 240 mIU/ml) at the end of observation. All four macaques receiving IM AAV had an increase in anti-HBs responses after the 3rd dose and all four had anti-HBs levels ≥10 mIU/ml (range 744 to 10,509 mIU/ml) at the end of observation. Treatment safety of dMNP delivery was assessed by visual checks at the immunization sites after dMNP application as well as a week after the vaccination. No animal from any group had any adverse reactions at the immunization sites.

### 3.4. HBsAg-specific CD4 and CD8 T cell responses

**Fig. 4.**
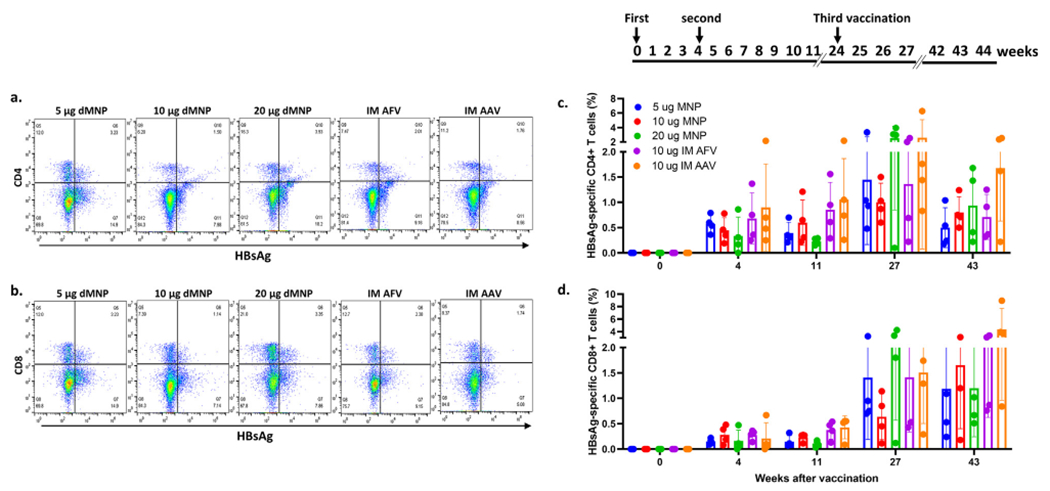
HBsAg-specific CD4+ and CD8+ T cell responses after vaccination by dMNP, IM AFV, and IM AAV of rhesus macaques. Flow cytometry of PBMC samples collected at 27 weeks from a representative animal receiving 5 μg dMNP, 10 μg dMNP, 20 μg dMNP, IM AFV, or IM AAV using APC-labeled HBsAg and PerCP-Cy5.5-anti-CD4 staining (**a**) and FITC-anti-CD8 staining (**b**). Vaccination schedule is shown on the top panel. (**c**) Percentage of HBsAg-specific CD4+ T cells in all vaccinated animals were determined by flow cytometry at 0, 4, 11, 27, and 43 weeks. (**d**) Percentage of HBsAg-specific CD8+ T cells were determined in all vaccinated animals by flow cytometry at 0, 4, 11, 27, and 43 weeks. Each data point represents an individual animal. Bars shown mean values ± standard deviation.

To determine whether HBsAg-specific T cell responses corresponded with anti-HBs levels in rhesus macaques, HBsAg-specific CD4+ and CD8+ producing T cells were measured in PBMCs by flow cytometry. T cell responses were analyzed at five different points, baseline (0 week), after the first HepB dose (4 weeks), after the 2nd HepB dose (11 weeks), after the 3rd HepB dose (27 weeks), and the end of observation (43 weeks). Flow cytometry results of HBsAg-specific CD4+ and CD8+ T cells at 27 weeks in the five different vaccination groups are shown in Fig. 4a and 4b. After the first HepB dose, HBsAg-specific CD4+ and CD8+ T cell responses were detected in all the vaccinated rhesus macaques regardless of anti-HBs levels (Fig. 4c and d). HBsAg-specific CD4+ and CD8+ T cell responses did not change after the 2nd dose. The 3rd HepB dose enhanced both HBsAg-specific CD4+ and CD8+ T cell responses. Frequency of HBsAg-specific CD4+ T cells peaked at 27 weeks in all five vaccination groups. HBsAg-specific CD8+ T cell responses were at a peak at the end of observation (43 weeks) in the 10 μg dMNP, IM AFV, and IM AAV groups. Notably, HBsAg-specific CD4+ and CD8+ T cells were detected not only in the macaques with protective levels of anti-HBs ≥ 10 mIU/ml, but also in the animals with anti-HBs levels below 10 mIU/ml. In addition, correlations between HBsAg-specific CD4+ or CD8+ T cells and anti-HBs levels were not observed in any of the vaccination groups (data not shown).

### 3.5. Transcriptional signature in the vaccination groups

**Fig. 5.**
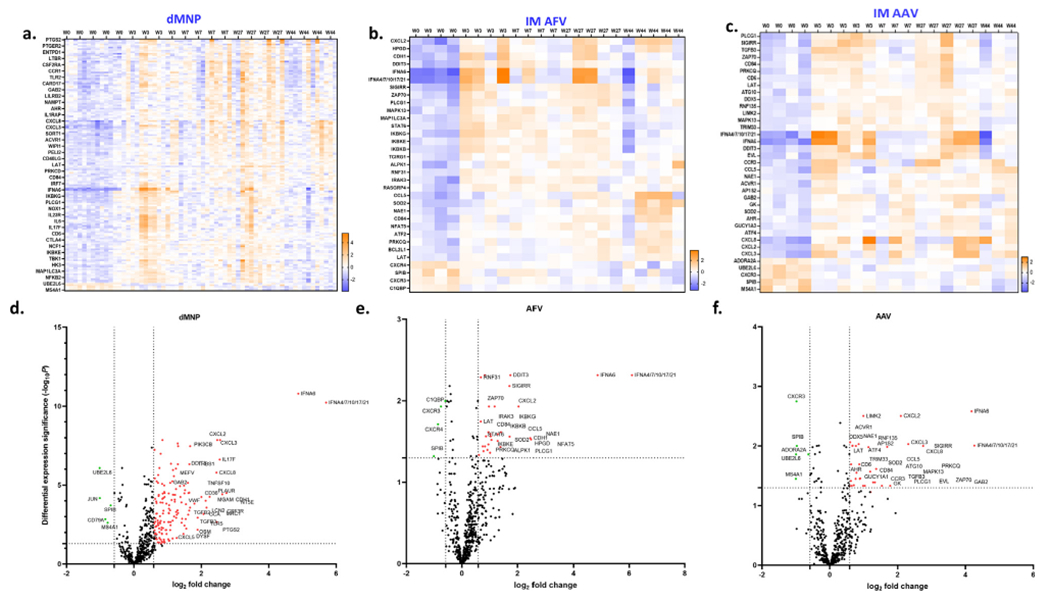
Transcriptional signature related to dMNP, IM AFV, and IM AAV vaccination in rhesus macaques. Heatmap showing differential expression of genes (DEGs) at 0, 3, 7, 27, and 44 weeks in the unified dMNP vaccination group (161 genes) (**a**), IM AFV group (33 genes) (**b**), IM AAV group (37 genes) (**c**). (**d**) Visualization of DEGs volcano plot of 785 genes (Host Response Profiling Panel). The representations are as follows: x-axis, log_2_Fold Change; y-axis, −log10 of a *P* value. The *P* values < 0.05 are in red dots and logFC ≥ 1.5 and logFC ≤ −1.5 are in green dots; the significant DEGs satisfying both criteria are in red and green dots and labeled with gene names. Black dots indicate the remaining genes present in the array that were not significantly significant. (For interpretation of the references to colour in this figure legend, the reader is referred to the web version of this article.)

MDS analysis of differentially expressed genes showed plots of baseline (control) samples were clustered together and separated from the vaccinated samples in the different vaccination groups (Supplemental Fig. 4). The patterns of proximities of gene expression in the 5 μg, 10 μg, or 20 μg dMNP vaccination groups were found to cluster together without segregation among these groups, indicating that similar patterns of gene expression were seen in these vaccination groups (Supplemental Fig. 4b). Based on this observation gene expression and pathway analysis for the three dMNP groups was performed as a single unified group. Differentially expressed genes and enriched pathways in the unified dMNP (n = 12), IM AFV (n = 4), and IM AAV (n = 4) vaccination groups were identified (Fig. 5 and Table 2). One hundred sixty-one genes (156 up-regulated and 5 down-regulated genes), 33 (29 up-regulated and 4 down-regulated genes) genes, and 37 (32 up-regulated and 5 down-regulated genes) genes were significantly regulated (fold change > 1.5, P < 0.05) as compared to baseline (week 0) samples in the dMNP, IM AFV, and IM AAV groups, respectively (Supplemental Tables 1, 2, and 3). The Heatmap of differentially expressed genes demonstrated that overall similar expression profiles were associated with vaccination group, but the levels of gene expression varied among the macaques in each vaccination group.

GSEA revealed that all three vaccination groups exhibited increases in NFκB signaling, T cell receptor signaling, and tissue stress (Table 2). Alpha-kinase 1 (ALPK1) signaling, IL-1 signaling, Nod-like receptor (NLR) signaling pathways, and DNA sensing were enriched pathways in both the dMNP and IM AFV groups. Peroxisome proliferator-activated receptors (PPAR) signaling, tumor necrosis factor (TNF) signaling, and RNA sensing were enriched pathways in both the dMNP and IM AAV groups. Chemokine signaling was an enriched pathway in both the IM AFV and IM AAV groups. dMNP vaccination induced increases in nitric oxide (NO) signaling, amyloide β binding, and GTPase activator binding. IM AFV vaccination induced mitogen-activated protein kinase (MAPK) and apoptosis. IM AAV vaccination induced increases in protein tetramerization, protein kinase R (PKR)-like endoplasmic reticulum kinase (PERK)-mediated unfolded protein response, and vasodilation. Cell types and tissues associated with differently expressed genes in vaccination groups were also identified using GESA. Differentially expressed genes in the dMNP group were shown to be expressed in CD33+ myeloid cells, CD14+ monocytes, the skin, and smooth muscle (Supplemental Table 4). In the IM AFV group, genes were expressed in CD8+ T cells, CD4+ T cells, dendritic cells, and ductal cells (Supplemental Table 5). Differentially expressed genes in the IM AAV group were expressed in eosinophils, microfold cells, and memory B cells. Eosinophils, memory B cells, T cells, blood, and immune system were associated with differentially expressed genes in the IM AAV group (Supplemental Table 6).

## 4. Discussion

In this study, we found that dMNP vaccination induced protective levels of anti-HBs antibody responses (≥10 mIU/ml) in mice and rhesus macaques at all three doses studied. No dose-dependent relationship for immunogenicity was seen in mice or rhesus macaques. The 5 μg dMNP induced higher anti-HBs levels compared to 10 μg or 20 μg dMNPs in mice but not in rhesus macaques (Figs. 4 and 6) suggesting that dose sparing could be used with these patches. This hypothesis is further supported by the finding that delivery of all three doses of dMNP induced higher anti-HBs responses compared to IM AFV vaccination in mice and rhesus macaques. In previous studies with vaccination by dMNP, dose sparing was reported for dMNP vaccination against rotavirus, influenza, and polio/rotavirus compared to IM vaccination in mice or rats [20,30,31]. However, poliovirus dMNP vaccine did not show dose sparing in rhesus macaques [32]. This is consistent with the broader literature, which suggests that dose-sparing often occurs when vaccinating in the skin by dMNP, other types of microneedle patches or intradermal injection, but not always [33,34].

dMNP vaccination induced higher anti-HBs antibody responses after the first HepB dose compared to IM AFV in mice (Fig. 2). After the second and third dose, anti-HBs antibody responses increased, and protective antibody responses were induced in all four vaccination groups in mice. In rhesus macaques, the IM AAV group induced protective levels of anti-HBs antibody responses after the first vaccine dose. Increased levels of anti-HBs antibody responses were detected in all rhesus macaques after the second and third vaccine doses. At the end of observation, two animals with 5 μg dMNP, all four animals with 10 μg dMNP, two animals with 20 μg dMNP, one animal with IM AFV, and all four animals with IM AAV had protective levels of anti-HBs antibody responses. These results indicated that dMNP vaccination induced better humoral immune responses than with IM AFV but was inferior to IM AAV in rhesus macaques. We found that delivery efficiency of the HBsAg by dMNP vaccination was found to be around 50 %. Therefore, the formulation used for dMNP fabrication should be improved to get the better delivery efficiency of the HBsAg and humoral antibody responses comparable to IM AAV. Though we found that two of four animals with either 5 μg or 20 μg dMNP had protective levels of anti-HBs responses, there was little variation in the amount of delivered antigen dose between animals with either dose (Fig. 3). These results suggest that the host immune responses to dMNP vaccination may react differently to induce antibody responses. Previous studies have shown that about 4 to 10 % of vaccinated infants or adults do not produce protective levels of antibody responses after the primary vaccination series (3 doses) [35–37]. Exact causes for non-responsiveness to hepatitis B vaccination are not clear. Factors like HLA haplotypes [38–40], insufficient or lack of T helper response [41,42] or inadequate antigen-presenting cell functions [40,43] were reported to be associated with low or absence of antibody response to HepB vaccination. A decline in anti-HBs titers was observed in some of the macaques in this study, which highlights that the duration of response needs to be addressed in future studies.

We found that HBsAg-specific CD4+ and CD8+ T cell responses were not different in dMNP vaccinated animals that exhibited either low or high anti-HBs responses. This was also true with the IM AFV group (Fig. 4). Correlation between T cell responses and humoral immune responses induced by HepB vaccine remains elusive as some studies [44,45] showed a correlation and others did not [46–48]. Our study demonstrated that T cell responses induced by HepB vaccination was not correlated with humoral immune response suggesting that another mechanism may be involved in low anti-HBs antibody responses to HepB vaccine.

In addition, average age and weights of rhesus macaques used in the current study was 1.9 year and 3.5 kg, respectively (1.6 to 2 years; range 2.6 to 5.1 kg). They were younger and lighter than those used for our previous study (average 6 years; 5 to 7 kg) [22]. A study showed that physiological age-dependent increase in antibody response in infancy where critical signal for B cell survival is poorly expressed in early-life bone marrow compartment [49]. The average weight of a newborn is 3.5 kg (range 2.5 to 4.5 kg). The rhesus macaques used in our previous study were larger and older than those used in our current study [21,22]. These differences may be additional factors for variable humoral immune responses detected in this study.

We analyzed profiles of differentially expressed genes as a transcriptional signature in each vaccination group. Tissue stress, T-cell receptor signaling, and NF-κB signaling pathways were enriched in all three vaccination groups (Table 1). The NF-κB signaling pathway is a major regulatory component to initiate inflammatory activation and adaptive immune responses in innate immune cells like macrophages, dendritic cells, monocytes, and neutrophils [50]. Diverse stimuli including cytokine pattern-recognition receptors (PRRs), T-cell receptor (TCR), and B-cell receptor can activate NF-κB signaling pathways [51]. Our observations provide evidence that HBsAg delivered either to the skin or by IM injection used similar mechanisms to induce innate immune and adaptive immune responses. Application of vaccine relies on presence of dendritic cells (DC) in tissue that take up the antigen, process it and present it to the T cells in the draining lymph node [52]. Epidermis and dermis in the skin contain more DCs such as Langerhans cells than those in muscle tissue [53].

We demonstrated that dMNP vaccination induced higher antibody responses compared to the IM AFV group in mice and rhesus macaques. Interestingly, CD33+ myloid cells and CD14+ monocytes were significantly associated with dMNP vaccination. Skin CD14+ is derived from CD14+ blood monocytes and prime CD4+ T cells into cells that induce naive B cells to switch isotype and become plasma cells [54,55]. Rhesus macaque CD33+ myloid cells showed effects on T cell inhibitory functions and these cells were also transiently increased after vaccination with influenza mRNA vaccine [56]. Another study showed that the gene expression profile of modified vaccinia virus Ankara (MVA) vaccine expressing HIV-1 subtype B antigen identified that cell types of CD33+ myloid cells and CD14+ monocytes were associated with humoral immune responses [57]. These observations in addition to our own suggest that CD33+ myloid cells and CD14+ monocytes may be induced as a result of inflammation and have a role in generating adaptive immune responses.

Some of the limitations of the current study include the small number of animals per vaccination group that were tested, which limited our ability to conduct statistical comparisons between vaccine groups. However non-human primates are an excellent experimental model system providing immune responses that resemble those of humans including infants [58]. Two animals with 5 μg or 20 μg dose dMNP exhibited no induction of protective levels of anti-HBs responses after three vaccinations; however, levels of HBsAg-specific CD4+ and CD8+ responses in these animals were comparable to the animals with the protective levels of anti-HBs responses. Future cellular immune response studies like analyzing levels of CD33+ and CD14+ T cells will be needed to determine low response levels of B cell responses in these animals.

To use dMNP in a public health setting, conducting human studies is required to show safety and efficacy of HepB-BD vaccination by dMNP and further validate the use of dMNP by minimally trained personnel in various settings outside of health care facilities.

In conclusion, we developed dMNPs to administer HBsAg and showed that delivery of vaccine by dMNP included robust humoral and cellular immune responses in mice and rhesus macaques as evidenced by anti-HBs levels comparable to seroprotective levels known to be induced by conventional IM delivery of the aluminum-adjuvanted vaccine in humans. In addition, dMNP vaccination induced anti-HBs antibody responses that were stronger than IM AFV vaccination, but weaker than IM AAV vaccination. Differentially expressed gene expression profiles indicated that similar innate and adaptive immune response mechanisms were induced to HBsAg delivered by dMNP, IM AFV, and IM AAV. These findings suggest that AFV delivery by dMNP could be effective for birth dose HepB vaccination in resource-limited regions, which could help end the hepatitis B epidemic by increasing vaccination access and coverage.

## 5. Data availability

The materials that support the findings of this study are available from the corresponding author upon request. All data needed to evaluate the conclusions in this paper are present in the paper or the Supplementary information.

## Supplementary Material

Supplementary Figure 3

Supplementary Figure 2

Supplementary Figure 1

Supplementary Figure 4

Supplementary Table 3

Supplementary Table 2

Supplementary Table 1 1

Supplementary Table 4

Supplementary Table 5

Supplementary Table 6

Supplementary figure legends

## Figures and Tables

**Table 1:** Vaccination groups used in mice and rhesus macaques.

	Group	Delivery type	dose (ug)
mice	group 1	dMNP	5
	group 2	dMNP	10
	group 3	dMNP	20
	group 4	IM AFV	10
rhesus macaques	group 1	dMNP	5
	group 2	dMNP	10
	group 3	dMNP	20
	group 4	IM AFV	10
	group 5	IM AAV	10

**Table 2:** Significantly enriched pathways, cell types, and tissues in dMNP, IM AFV, and IM AAV groups after hepatitis B vaccination.

dMNP Group	Significance Score (−Log_10_*P*)	IM AFV Group	Significance Score (−Log_10_*P*)	IM AAV Group	Significance Score (−Log_10_*P*)
Enrichment Pathways		Enrichment Pathways		Enrichment Pathways	

ALPK1 Signaling	4.96	cytokine-mediated signaling pathway	2.87	protein tetramerization	3.55
PPAR Signaling	4.82	Tissue Stress	2.64	mRNA transcription	3.02
NO Signaling	4.35	MAPK Signaling	2.48	PERK-mediated unfolded protein response	2.73
RNA Sensing	4.04	DNA Sensing	2.47	vasodilation	2.73
DNA Sensing	3.97	mitochondrial matrix	2.47	store-operated calcium entry	2.65
NLR Signaling	3.96	phospholipase activity	2.46	leucine zipper domain binding	2.64
Tissue Stress	3.93	Apoptosis	2.44	Tissue Stress	2.53
T cell receptor Signaling	3.90	NLR Signaling	2.32	TGF-beta Signaling	2.32
IL-1 Signaling	3.69	COP9 signalosome	2.29	PPAR Signaling	2.28
NF-κB transcription factor activity	2.91	mitotic DNA integrity checkpoint	2.29	Chemokine Signaling	2.27
macrophage cytokine production	2.69	Proteotoxic Stress	2.25	NF-κB Signaling	2.25
cellular response to hydrogen peroxide	2.65	ALPK1 Signaling	2.22	glutamate receptor binding	2.18
release of cytochrome *c* from mitochondria	2.58	IL-1 Signaling	2.22	Phagocytosis	2.17
protein heterodimerization activity	2.41	Chemokine Signaling	2.20	RNA Sensing	2.17
TNFβ binding	2.08	T cell receptor signaling pathway	2.11	SMAD binding	2.14
amyloid-beta binding	2.08	response to toxic substance	2.07	protein heterodimerization activity	2.12
GTPase activator activity	2.02	immunological synapse	1.82	TNF Signaling	2.11
peptidoglycan binding	2.02	cell–cell junction	1.80	T cell receptor Signaling	2.11
		IκB kinase complex	1.79		

Cell Types and Tissues		Cell Types and Tissues		Cell Types and Tissues	

CD33+ Myeloid	5.54	CD8+ T cells	0.89	Eosinophils	0.64
CD14+ Monocytes	1.61	CD4+ T cells	0.89	Microfold cells	0.64
Whole Blood	0.96	Plasmacytoid dendritic cells	0.80	B cells memory	0.64
Skin	0.61	Airway epithelial cells	0.57	Ductal cells	0.64
Connective tissue	0.61	Ductal cells	0.57	T cells	0.64
Lungs	0.61	Epithelial cells	0.57	Lungs	0.53
GI tract	0.61	Eosinophils	0.57	Blood	0.53
Neutrophils	0.41	Blood	0.50	GI tract	0.53
Platelets	0.41	Epithelium	0.50	Pancreas	0.53
Smooth Muscle	0.39	Lungs	0.50	Immune system	0.53
pineal day	0.39	Heart	0.50	CD4+ T cells	0.44
Satellite glial cells	0.32	Thyroid	0.45	CD8+ T cells	0.41

## Data Availability

Data will be made available on request.
