# Supplementary figures and images for: Hepatitis B vaccine delivered by microneedle patch: Immunogenicity in mice and rhesus macaques
Source: Vaccine. Author manuscript; Available in PMC 2024 Mar 25. (PMC10961677; doi:10.1016/j.vaccine.2023.05.005)

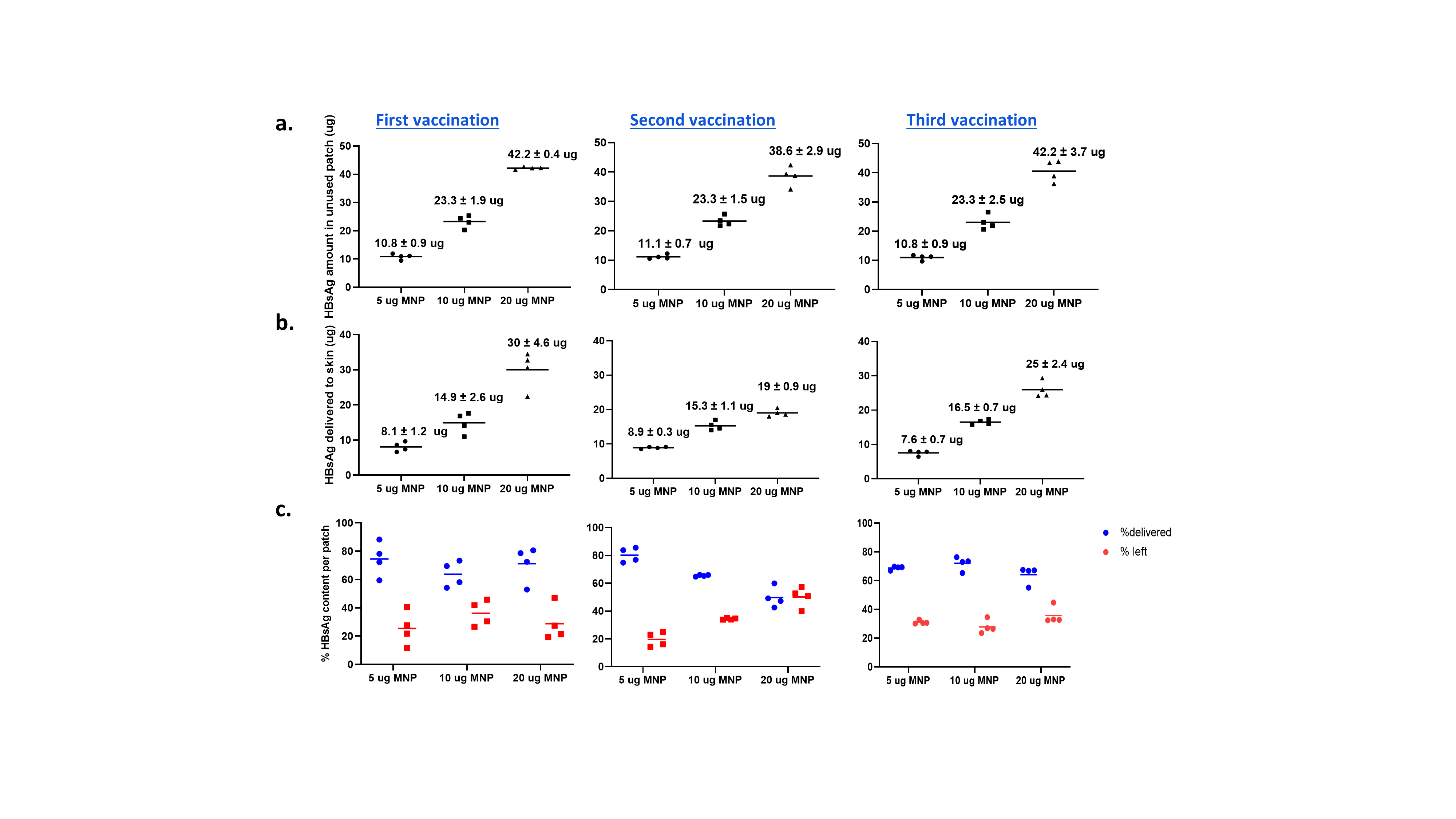

Supplement: Supplementary Figure 3 [file NIHMS1972829-supplement-Supplementary_Figure_3.tif]

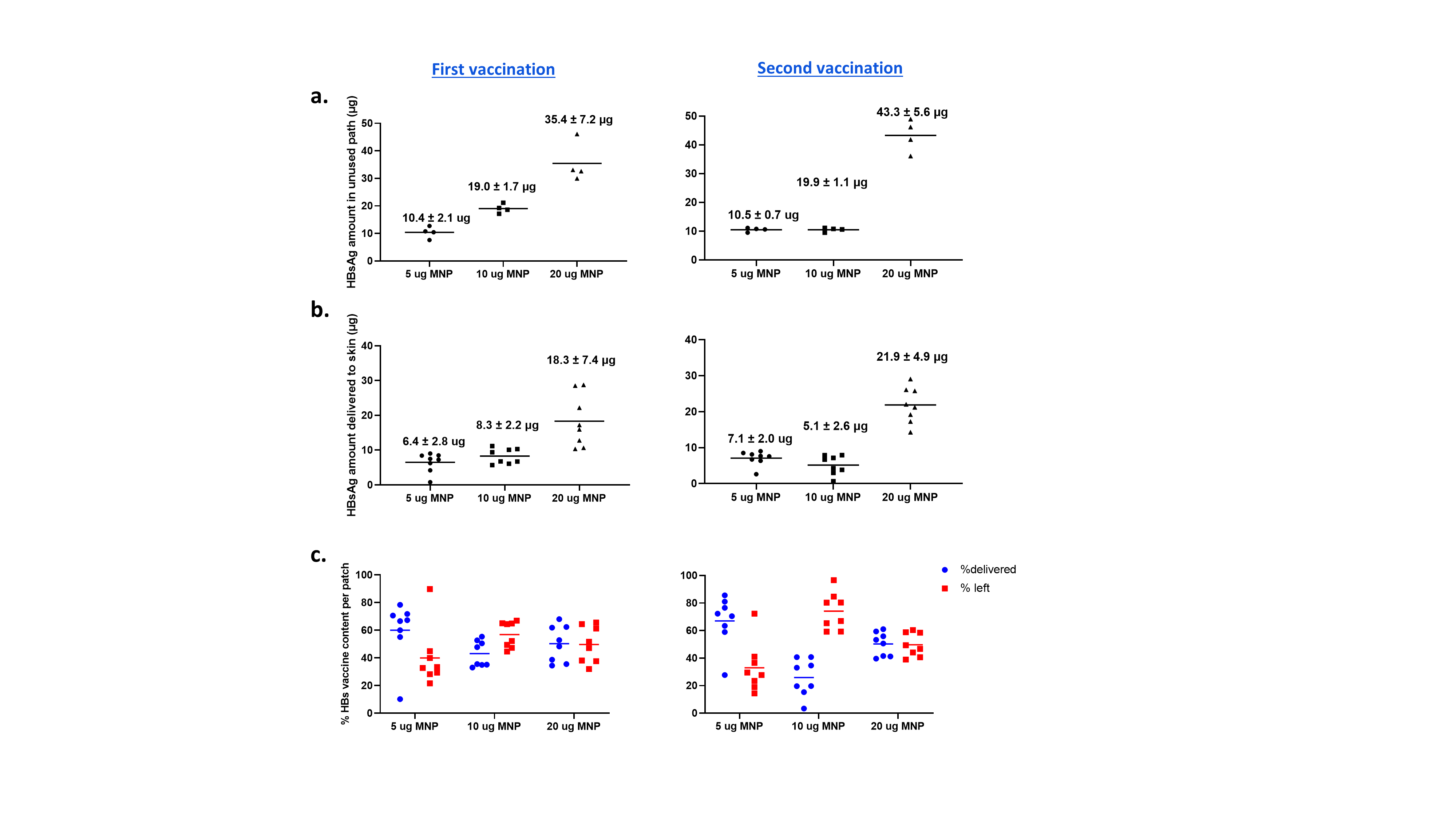

Supplement: Supplementary Figure 2 [file NIHMS1972829-supplement-Supplementary_Figure_2.tif]

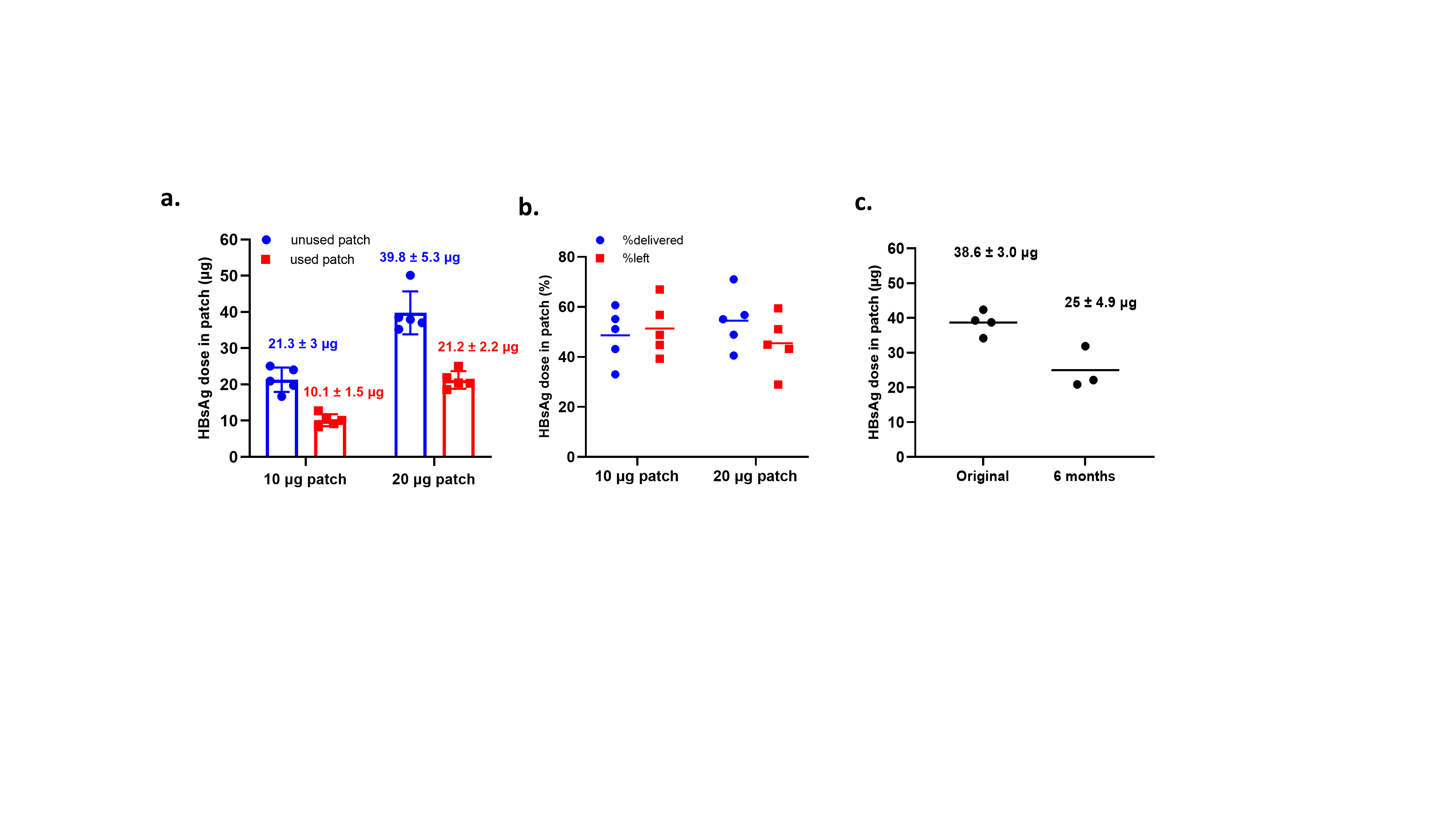

Supplement: Supplementary Figure 1 [file NIHMS1972829-supplement-Supplementary_Figure_1.tif]

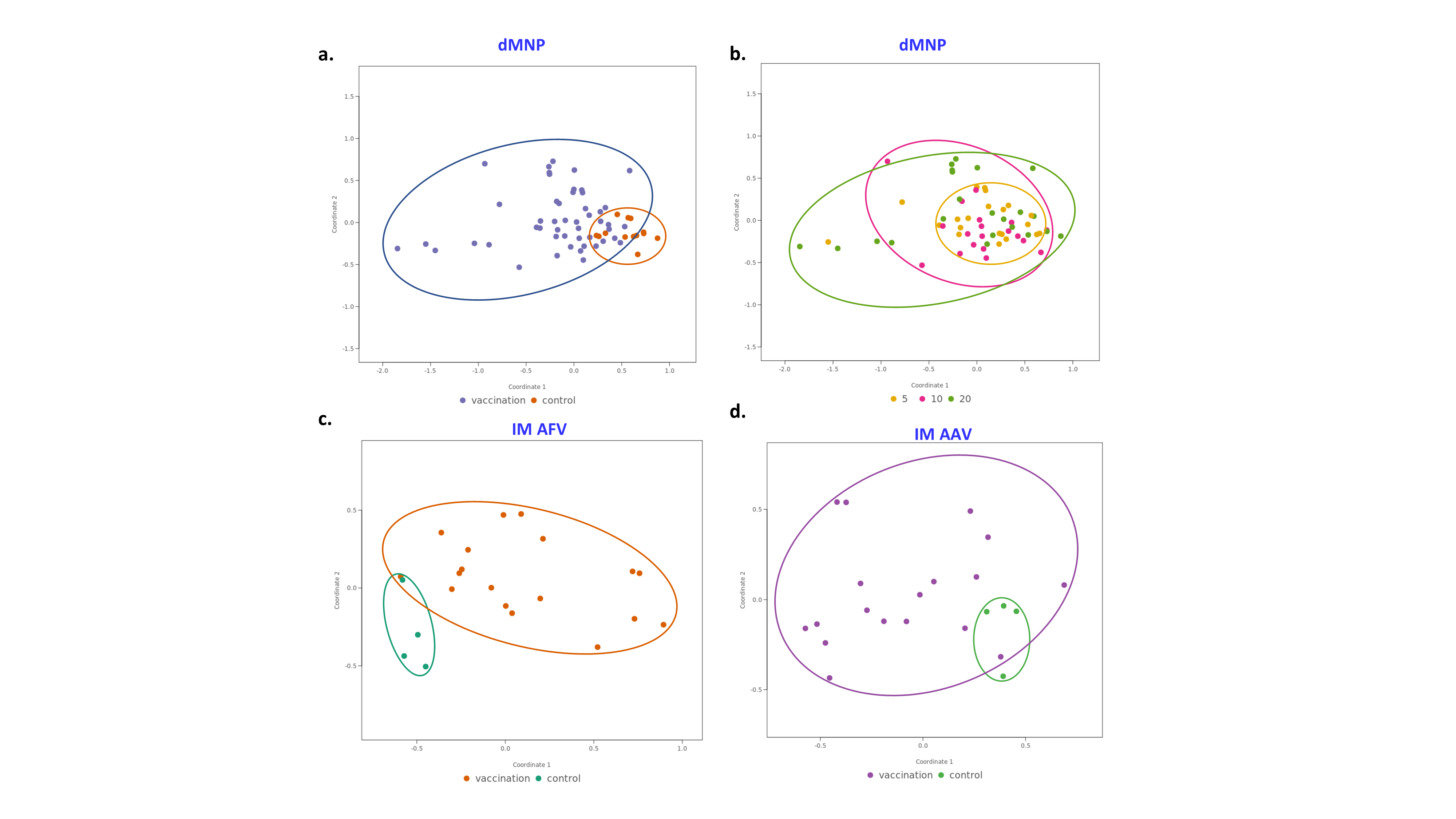

Supplement: Supplementary Figure 4 [file NIHMS1972829-supplement-Supplementary_Figure_4.tif]
