# Supplementary material for: Hepatitis B vaccine delivered by microneedle patch: Immunogenicity in mice and rhesus macaques
Source: Vaccine. Author manuscript; Available in PMC 2024 Mar 25. (PMC10961677; doi:10.1016/j.vaccine.2023.05.005)
Supplement: Supplementary figure legends [file NIHMS1972829-supplement-Supplementary_figure_legends.docx]

**Supplementary figure legends**

**Supplemental Figure 1. HBsAg dose delivered by dMNPs into porcine skin *ex vivo***. (**a**) HBsAg dose in unused and used dMNPs designed to administer 10 µg or 20 µg HBsAg (n=5 per group). After applying in pig skin *ex vivo,* residual amounts of HBsAg on used dMNPs (red bars) were compared to those in the unused dMNPs (blue bars). Data shown mean values ± standard deviation. (**b**) HBsAg delivery expressed as a percentage of vaccine dose initially loaded into dMNPs (n=5 per group), which was calculated by subtracting residual amount of HBsAg in the used patch from HBsAg amount in the unused patch. (**c**) Stability of dMNPs was tested after storing at room temperature (20 to 25 ^0^C) for 6 months. Horizontal bars shown mean values.

**Supplemental Figure 2. HBsAg dose delivered by dMNPs to mice in vivo**. (**a**) Three doses (5 µg, 10 µg, 20 µg) of dMNP were prepared and HBsAg amounts were measured in the original (unused) patches (n=4 per group). (**b**) After the first and second vaccine dose, residual amounts of HBsAg in the used patches was measured and delivered HBsAg amounts were calculated by subtracting the residual HBsAg amounts in the used patches from HBsAg amounts in the original (unused) patches (n=4 per group). Delivered HBsAg from each dMNP dose are shown in x-axis.

**Supplemental Figure 3. HBsAg dose delivered by dMNPs to rhesus macaques in vivo**. (**a**) Three doses (5 µg, 10 µg, 20 µg) of dMNP were prepared and HBsAg amounts were measured in the original (unused) patches (n=4 per group). (**b**) After the first, second, and third vaccine dose, residual amounts of HBsAg in the used patches was measured and delivered HBsAg amounts were calculated by subtracting the residual HBsAg amounts in the used patches from HBsAg amounts in the original (unused) patches (n=4 per group). (**c**) Delivered HBsAg from each dMNP dose is shown as a percentage of HBsAg content in the patch as a measure of delivery efficiency (n=4 per group). Data shown mean values ± standard deviation.

**Supplemental Figure 4. Multidimensional scaling (MDS) analysis of differently expressed gene expression data in rhesus macaques.** Each dot represents an individual sample in the unified dMNP, IM AFV, and IM AAV groups. (**a**) samples are segregated as baseline samples versus vaccinated samples in the unified dMNP group, (**b**) samples are segregated as 5 µg dMNP, 10 µg dMNP, 20 µg dMNP samples, (**c**) samples are segregated as baseline samples versus vaccinated samples in the IM AFV group, (**d**) samples are segregated as baseline samples versus vaccinated samples in the IM AAV group. Coloring for the sample data dots is shown below each panel. Clustering of groups is shown with ellipses colored as the group they encircle.
